# TacticUP Video Test for Soccer: Development and Validation

**DOI:** 10.3389/fpsyg.2020.01690

**Published:** 2020-08-04

**Authors:** Guilherme Machado, Israel Teoldo da Costa

**Affiliations:** Physical Education Department, Centre of Research and Studies in Soccer (NUPEF), Universidade Federal de Viçosa, Viçosa, Brazil

**Keywords:** tactical skill, tactical awareness, talent development, decision-making, video-based test, perceptual-cognitive skill

## Abstract

This study aims to expand the process of tactical assessment of soccer players through the development and validation of a video-based test based on core tactical principles of play. The TacticUP video test for soccer is composed of offensive and defensive video sequences of 11vs11 soccer situations. Participants should choose the most appropriate solution for each video sequence. Content validity was established based on a panel of nine experts from four different countries. Construct validity was assessed through the comparison between players with distinct expertise levels. Comparisons between groups’ final scores showed statistical differences (*p* < 0.05) in 10 out of the 15 variables assessed, in which the expert group displayed higher values compared to the non-expert group. Face validity examined the acceptability and suitability of the test by players. Reliability was determined through the test–retest method for each video sequence, and Cohen’s Kappa values ranged from 0.622 to 1.0. Therefore, the TacticUP video test showed adequate content, construct, and face validity and was a reliable measure of perceptual-cognitive and decision-making skills in soccer. We overcame limitations from previous video-based tests in soccer by introducing situations concerning off-the-ball movements in both offensive and defensive phases. The practical applications of this test are: (i) it can be used to monitor players’ perceptual-cognitive and decision-making skills; (ii) the test is based on players’ response selection in a video-based test, which enables the measurement of their perceptual-cognitive and decision-making skills based on the core tactical principles of play; (iii) generate players’ tactical profile considering their perceptual-cognitive and decision-making skills based on core tactical principles of play; and (iv) measure the effectiveness of intervention/training programs on the development of perceptual-cognitive and decision-making skills.

## Introduction

Making efficient decisions is an essential feature for athletes to achieve superior performance in sports (Gréhaigne et al., 2001). Decision-making skills are related to both perceptual-cognitive and perceptual-motor skills. Perceptual-cognitive skill can be considered to be what one is able to perceive and understand; perceptual-motor skill refers to what one is able to perceive and do through movement (Marteniuk, 1976; Starkes et al., 2004). In this context, decision-making can be defined as an action choice, and it is an outcome that can be observed as a motor or verbal response (Macmahon and Mcpherson, 2009; Bruce et al., 2012). The decision-making skill is based on players’ game reading skill, which can be defined in soccer as the players’ ability to notice and integrate the moving information on the field, including the ball, teammates, opponents, as well as the actions they perform (Williams, 2000; Hartigh et al., 2017). Furthermore, decision-making skill is also related to sport-specific knowledge, which is defined by Thomas et al. (1986, p. 259) as “…a complex product of cognitive knowledge about current situation and past events combined with a player’s ability to produce the sport skill(s) required.” The sport-specific knowledge may range from a response selection (perceptual-cognitive skills) to a response execution component (perceptual-motor skills) and is closely related to the tactical domain in individual and collective sports (McPherson, 1994; Williams, 2000; Starkes et al., 2004; Macmahon and Mcpherson, 2009; Bonney et al., 2019).

In soccer, tactical knowledge is considered an important factor for players to achieve high performance, considering that every action of the game has a tactical purpose (Garganta, 2009). Teoldo et al. (2017, p. 26) define tactics as “…the management (positioning and displacement/movement) of the playing space by players and teams.” As for the concept of tactical knowledge, it has been divided into two distinct forms, namely, declarative (DTK) and procedural tactical knowledge (PTK) (Anderson, 1983; McPherson, 1994). On the one hand, DTK is related to the perceptual-cognitive skills and refers to the knowledge about the rules and objectives of the game linked to the awareness of response selection (i.e., “knowing what to do”). On the other hand, PTK is related to the perceptual-motor skills and refers to the response selection and execution in game situations (i.e., “doing it”) (McPherson, 1994; Starkes et al., 2004). Both types of skills can be understood as a continuum, which includes possibilities ranging from “knowing what do” to “doing it” (McPherson, 1994; Williams, 2000; Starkes et al., 2004).

Considering this continuum, there is evidence that both perceptual-cognitive skills and perceptual-motor skills are related to soccer players’ development over time (Teoldo et al., 2010a; Américo et al., 2017), superior future performance (Aquino et al., 2017), playing position (Williams et al., 2008), technical skills (Aquino et al., 2016; Rechenchosky et al., 2017b), and different levels of expertise (Roca et al., 2012). As for the topic of expertise, previous research demonstrated that both perceptual-cognitive skills and perceptual-motor skills increase according to the level of expertise (Mesquita et al., 2012; Roca et al., 2012). Despite the indications about the important role of perceptual skills and tactical knowledge plays in players’ performance, the current instruments have some limitations (Teoldo et al., 2017).

Some of the aforementioned limitations regarding the currently available instruments for assessing DTK are related to their methodology. One such instrument, developed by Elferink-Gemser et al. (2004), is based on a self-assessed (perceived) tactical skills questionnaire and raised questions about the scientific control of such measures as it relies on subjective assessment of players’ self-perception (Nortje et al., 2014). Another instrument is a video-based test developed by Mangas (1999). This test is based on analysis of official matches video sequences, where participants have to choose the best solution for each situation and are instructed to answer, “What should the player in possession of the ball do?” However, this test only assesses the offensive phase and is based on the analysis of situations that take into account the actions of the player with the ball. Consequently, test results are unable to inform players’ perceptual-cognitive skills based on off-the-ball situations, since during the game, players spend between only 1 and 3% of the time in possession of the ball (Garganta, 1997). This implies that the knowledge about players’ actions and movements performed without the ball – which accounts for 97% of the total playing time – is not assessed by this test and thus represents a serious limitation. Hence, the aforesaid limitations may be understood as a gap on current DTK and perceptual-cognitive skills assessment protocols that needs to be overcome with more coherence and representativeness with respect to the correspondence between what is being measured and what players actually perform in actual match contexts.

In order to achieve this coherence, an important aspect of this type of assessment to be taken into account is its theoretical construct basis (González-Víllora et al., 2015). The assessment needs be grounded on proposals that consider the logic of the game, which allows the evaluation of players’ knowledge and perceptual skills regarding the management of playing space. Additionally, consonance between training and tactical assessment on a regular basis is key to qualify the process of talent identification and development (Gréhaigne et al., 2001; Sarmento et al., 2018). In this regard, the assessment based on tactical principles meets both criteria once they were designed based on the logic of the game (Teoldo et al., 2010) and those, consequently, are the contents on which the training of tactical skills is grounded (Teoldo et al., 2017). Fortunately, an instrument was developed based on such assumptions, although used for assessing perceptual-motor skills, through the player’s tactical behavior, which is the System of Tactical Assessment in Soccer (FUT-SAT) (Teoldo et al., 2011b). Therefore, considering the tactical continuum that ranges from “knowing what do” to “doing it,” coherence in tactical skill assessment can be achieved by developing a perceptual-cognitive and decision-making skill test based on the same theoretical background of FUT-SAT (i.e., core tactical principles of play) (McPherson, 1994; Williams, 2000; Starkes et al., 2004; Bonney et al., 2019).

These principles represent “a set of ground rules that guide players and teams’ actions in both phases of play (defense and attack), in order to create imbalances in the opponent’s organization, stabilize the organization of the team and provide players with an adjusted intervention within the center of play” (Teoldo et al., 2009, p. 2). The development of a perceptual-cognitive and decision-making skills assessment instrument based on such principles will enable a more effective assessment of tactical skill based on the complementarity of both types of knowledge considering the tactical continuum (McPherson, 1994; Williams, 2000; Starkes et al., 2004; Bonney et al., 2019). Additionally, it will support research based on theory-driven assumptions (Anderson, 1983; McPherson, 1994; Williams, 2000; Starkes et al., 2004). In order to preserve the representativeness of the actual game, the assessment of players’ knowledge and perceptual-cognitive and decision-making skills should be performed in more representative tasks, which can enhance its transferability and applicability in competitive contexts. In this regard, the use of video-based tests is preferable once they provide a more natural perception of the scene when compared to static figures or questionnaires (Mann et al., 2007). Therefore, the purpose of this study is to expand the process of tactical assessment of soccer players through the development and validation of a video-based test based on the core tactical principles of play.

## Materials and Methods

### Ethics Statement

This study was conducted according to the ethical guidelines of the lead institution, the standards of the Declaration of Helsinki (2008), and the National Health Council (2012). Participants and their parents signed a legal consent authorizing the collection of data and their utilization for research purposes.

### Development and Validation of the TacticUP Video Test

The development and validation of the TacticUP video test for soccer followed the procedures suggested by Cronbach (1988) and took into account important characteristics indicated by literature, such as: (i) acceptability of the test among the individuals who took the test (face validity), (ii) the extent to which a measure represents a construct (content validity), (iii) allowing to distinguish individuals with different skill levels (construct validity), and iv) the consistency and repeatability of measurements (reliability) (Cronbach and Meehl, 1955; Landis and Koch, 1977; Anastasi, 1988; Cronbach, 1988; Gréhaigne et al., 1997; Hopkins, 2000). With such characteristics, the instrument allows measuring perceptual-cognitive and decision-making skills in different contexts.

### Structure of the Test

**FIGURE 1:**
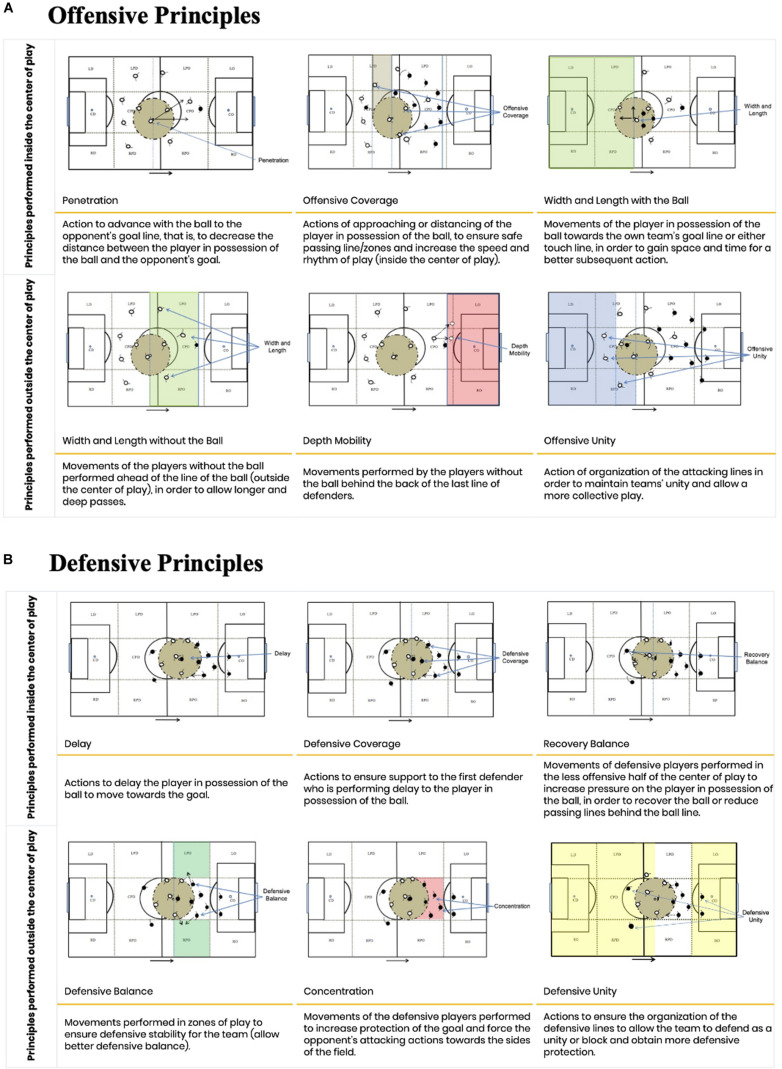
Description of the core tactical principles of soccer: **(A)** offensive and **(B)** defensive (Source: Teoldo et al., 2015).

The TacticUP video test for soccer was based on the core tactical principles of soccer (Teoldo et al., 2009, 2017). These principles enable players to find effective solutions for game situations through the management of playing space inside and outside the center of play (Teoldo et al., 2017). The center of play is a circumference of 9.15 m radius from the location of the ball. It was conceived based on the official laws of soccer as it is assumed that players located farther than 9.15 m from the player in possession of the ball cannot interfere directly in his actions (Teoldo et al., 2017). The principles are categorized according to the phase of play, namely: (i) penetration, (ii) offensive coverage, (iii) depth mobility, (iv) width and length, and (v) offensive unity for the offensive phase. For the defensive phase, the principles are: (vi) delay, (vii) defensive coverage, (viii) balance, (ix) concentration, and (x) defensive unity (Figure 1). These principles were proposed because they display central aspects of the educational process of tactical skills. Furthermore, these principles possess objective measures of players’ movements related to the management of the playing space.

The TacticUP video test for soccer comprises offensive and defensive video sequences (scenes) of 11 vs. 11 soccer situations. The videos were recorded from a bird’s-eye view, which is an elevated perspective of an object from above. This perspective was chosen because it allows players to visualize the tactical principles whether they were performed close or far from the ball. The test is composed by scenes of each tactical principle from both offensive and defensive phases. In relation to the offensive principle of width and length, scenes were designed for “width and length with the ball” and “width and length without the ball.” The same procedure was followed for the defensive principle of balance, as scenes were designed for both “recovery balance” and “defensive balance.” This procedure was carried out following the update of these tactical principles, proposed by Teoldo et al. (2017), which represent differences on the management of playing space for each of these tactical principles. For each scene, participants had to choose the most appropriate solution from four possible scenarios. Prior to the start of the test, instructions were given to participants with respect to the structure of the test, and three practice trials were presented in order to familiarize participants to the task. These three scenes included two offensive sequences (one scene with the observed player with the ball and the other scene with the observed player without the ball) and one defensive sequence (the observed player’s team was in defensive phase). These three conditions were chosen to enable participants’ awareness about the nature of the video sequences they were going to watch. These scenes were edited through the utilization of the software Videobserver and iMovie. The test is currently available at www.tacticup.com.br.

**FIGURE 2:**
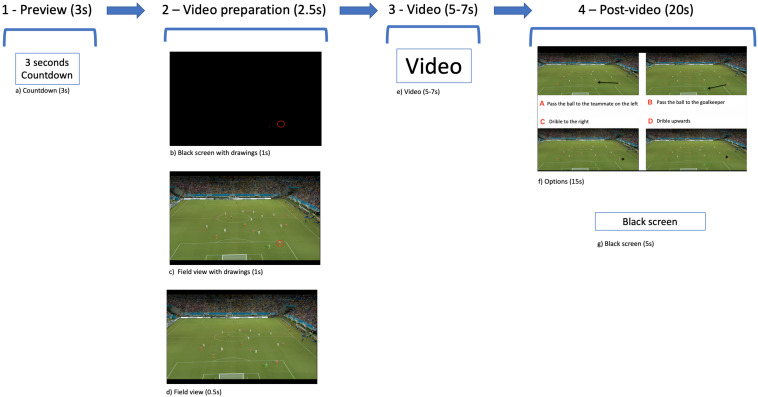
Order of presentation of the scenes.

The scenes were presented in the following order (Figure 2): (a) a 3-s countdown is displayed onto the screen; (b) the screen turns black, and a red dot representing the place where the ball will appear and a red circle on the location where the observed player will appear are shown; (c) a static image of the field is shown with the red dot and circle markings in order to identify the ball and the observed player; (d) the red dot and circle markings disappear, and only the static image of the field is shown; (e) the video scene starts; (f) response options are shown (A, B, C, and D), and participants choose the most appropriate option for that given situation; and (g) finally, a black screen is shown. The video scenes are occluded before the evolving game play is concluded (e.g., player in possession of the ball making a pass, dribbling forward, or shooting at goal). At this point, participants are asked to respond to a queue question “what the observed player should do?” by filling in the answer sheet. This sequence was identical for every scene of the test. Participants watched three scenes of each tactical principle. All the scenes included in the test met the inclusion criteria for its content validity (described in section “Content Validity”).

The score for each scene was calculated based on the correspondence between participants’ responses and those chosen by a panel of experts (*n* = 9). The final scores provided by the TacticUP video test for soccer are presented in 15 items, one for each core tactical principle, in addition to scores for the offensive phase, defensive phase, and game (offensive and defensive phases together).

### Content Validity

The selection and edition of the scenes were based on conceptual descriptions and objective parameters for representation of the core tactical principles (Teoldo et al., 2009, 2017). In this regard, the selection of the scenes was carried out considering: (i) the spatial references for the performance of core tactical principles (Teoldo et al., 2017) and (ii) the occurrence, within the game, of the performance indicator(s) of each core tactical principle (Teoldo et al., 2017). For instance, with respect to the selection of a scene that displays the principle of penetration, which is related to actions of progressing with the ball toward the opponent’s goal line, the following criteria should be met: (i) the observed player must be in possession of the ball, progressing toward the opponent’s goal line; (ii) the outcome of this action is one of the performance indicators of this tactical principle (e.g., allowing the player to pass the ball to a teammate or to shoot at goal) (Teoldo et al., 2017).

For measurement of content validity of the scenes, nine experts from four different countries participated in this study, namely, Brazil, United Kingdom, sPortugal, and Spain. An expert was considered a professional with 10 or more years of experience (Ericsson et al., 1993) in soccer (as a coach or academic), which is in agreement with the concept established by Ericsson et al. (2006) that considers an expert as a very skillful and recognized individual in a specific domain, who achieved a good level of expertise through deliberate practice. Experts ranked the response options for each scene as first, second, third, and fourth most adequate for that situation. Additionally, they were encouraged to provide feedback regarding the appropriateness of the content and context of the videos and the response options in each scene, as suggested by previous research (Larkin et al., 2014). As inclusion criteria, the responses regarding the situations presented on the scenes should have an agreement above 70% among experts with respect to the best option for each scene, similarly to previous research (Natsuhara et al., 2020), which represented the inclusion of 63% of the overall number of scenes assessed by the experts.

### Construct Validity

Construct validity is related to the power of a given test to distinguish individuals with distinct performance/characteristics (Anastasi, 1988; Thomas et al., 2011). In this regard, we assessed 149 soccer players between 11.0 and 17.9 years of age (14.9 ± 1.6 years) from three soccer clubs. All participants were engaged in training routines with at least three weekly sections and participated in competitions at the national level for their age groups, in addition to being affiliated to their respective state soccer federations.

For assessment of construct validity, two groups of different expertise levels were compared, according to the amount of accumulated training hours, assessed through a validated retrospective questionnaire (Ward et al., 2007). This categorization was carried out taking into account theoretical assumptions (Ericsson and Smith, 1991) and empirical data that indicate that the amount of accumulated training hours is a discriminating factor between different levels of expertise (Ward et al., 2007; Weissensteiner et al., 2008; Ford et al., 2009, 2010; Roca et al., 2012). Players were ranked according to accumulated training hours and 25% of the top, the high training hours (HTH) group (*n* = 38, *M* = 2.019.0, *SD* = 789.4 h of training, 16.5 ± 1.0 years of age) were considered for analysis, as well as 25% of the bottom, the low training hours (LTH) group (*n* = 38, *M* = 95.8, *SD* = 95.6 h of training, 14.1 ± 1.7 years of age). The remainder of the sample was excluded from analysis. This procedure was used to ensure that the procedure for grouping participants of different levels of expertise (based on accumulated training hours) was based on objective criteria that statistically distinguished both analyzed groups (*p* < 0.001), as carried out in previous research (Ford et al., 2010; Roca et al., 2012; Williams et al., 2012). Although the age of the groups HTH and LTH was slightly different, previous research showed that early experience in soccer, rather than age, is the moderate factor that influences tactical knowledge and perceptual-cognitive and decision-making skills (Kannekens et al., 2009; Roca et al., 2012; Serra-Olivares, 2014; Serra-Olivares and García-López, 2016). Subsequently, we verified the between-group differences in all the final scores through independent *t*-test and Mann–Whitney *U* test. Distributions were checked for normality with a Kolmogorov–Smirnov test. The effect size for the Mann–Whitney tests was calculated through the formula described by Fritz et al. (2012). The interpretation of *r*-value was made as follows (Ferguson, 2009): no effect 0–0.19; minimum effect 0.20–0.49; moderate effect 0.50–0.79; and strong effect >0.80. The effect size for the independent *t*-tests was calculated through the Cohen’s d value, and the interpretation was made as follows (Cohen, 1992): no effect 0–0.19; minimum effect 0.20–0.49; moderate effect 0.50–0.79; and strong effect >0.80. Statistical procedures were performed through SPSS 22.0.

### Face Validity

Face validity was determined through participants’ responses for two questions at the end of the test in order to verify acceptability and suitability of the test, according to participants’ motivation to perform the task (Anastasi, 1988). The questions were: (Q1) “Did you enjoy taking the test?” and (Q2) “If asked, would you like to take the test again?” (Table 1).

**TABLE 1:** Players’ acceptability values for the TacticUP video test for soccer.

**(Q1) Did you enjoy taking the test?**		**(Q2) If asked, would you like to take the test again?**	
**Yes**	**No**	**Yes**	**No**
140 (94.0%)	9 (6.0%)	125 (83.9%)	24 (16.1%)

### Reliability

Reliability was verified through the test–retest method (Baumgartner and Jackson, 1991), respecting the interval of 21 days, to avoid familiarity with the task (Robinson and O’Donoghue, 2007). Fifteen players, representing 10.1% of the sample, were reassessed, according to the minimum value (10%) recommended by literature (Tabachnick and Fidell, 2013). Cohen’s Kappa was used to determine the reliability of each scene between the first and second applications of the test. The categorization of Kappa values followed the reliability scale established by Landis and Koch (1977): poor agreement (<0.00), slight agreement (0.00–0.20), fair agreement (0.21–0.40), moderate agreement (0.41–0.60), substantial agreement (0.61–0.80), and almost perfect agreement (0.81–1.00). Kappa values of the scenes (*M* = 0.823, SE = 0.095) ranged from 0.622 to 1.0, which means that they were classified between “substantial” and “almost perfect” agreement.

## Results

### Construct Validity

**FIGURE 3:**
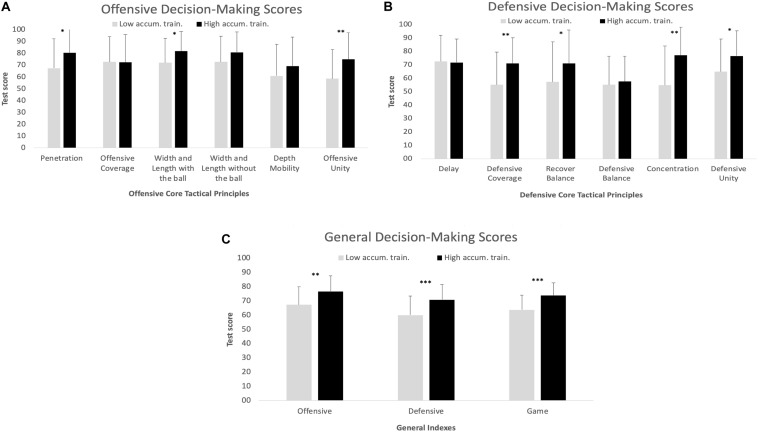
Comparison of test scores between groups with low accumulated training hours and high accumulated training hours in **(A)** Offensive Decision-Making Scores, **(B)** Defensive Decision-Making Scores, and **(C)** General Decision-Making Scores. *p < 0.05, **p < 0.01, ***p < 0.001.

Comparisons between groups’ final scores displayed statistical differences for 10 out of 15 variables assessed, whereas the HTH displayed higher values compared to LTH for all such variables (Figure 3). With respect to the core tactical principles, differences were found for the principles of: (i) penetration, in which HTH (*M* = 80.3, *SD* = 20.2) scored higher than the LTH (*M* = 67.4, *SD* = 24.7), *U* = 506.00, *z* = -2.26, *p* = 0.023, *r* = -0.258, *minimum effect*; (ii) width and length without the ball, in which HTH (*M* = 81.8, *SD* = 16.5) outscored the LTH (*M* = 72.0, *SD* = 20.7), *U* = 518.00, *z* = -2.13, *p* = 0.033, *r* = -0.243, *minimum effect*; (iii) offensive unity, in which the HTH (*M* = 74.9, *SD* = 22.5) outscored the LTH (*M* = 58.5, *SD* = 24.8), *U* = 449.00, *z* = -2.85, *p* = 0.004, *r* = -0.325, *minimum effect*; (iv) defensive coverage, in which HTH (*M* = 70.9, *SD* = 19.1) scored higher than the LTH (*M* = 55.2, *SD* = 24.2), *U* = 427.00, *z* = -3.07, *p* = 0.002, *r* = -0.350, *minimum effect*; (v) defensive balance, in which the HTH (*M* = 70.9, *SD* = 24.9) scored higher than the LTH (*M* = 57.3, *SD* = 29.8), *U* = 529.00, *z* = -2.03, *p* = 0.042, *r* = -0.231, *minimum effect*; (vi) concentration, in which the HTH (*M* = 77.0, *SD* = 21.0) outscored the LTH (*M* = 54.9, *SD* = 29.2), *U* = 392.00, *z* = -3.46, *p* = 0.001, *r* = -0.394, *minimum effect*, and (vii) defensive unity, in which HTH (*M* = 76.6, *SD* = 18.8) outscored the LTH (*M* = 64.9, *SD* = 24.3), *U* = 518.50, *z* = -2.13, *p* = 0.033, *r* = -0.243, *minimum effect*.

With respect to the general indexes (Figure 3), in the offensive phase, the HTH (*M* = 76.7, *SD* = 10.8) scored higher than the LTH (*M* = 67.3, *SD* = 12.5), *t*(74) = -3.33, p = 0.001, *d* = 0.804, *strong effect*; in the defensive phase, the HTH (*M* = 70.8, *SD* = 10.7) outscored the LTH (*M* = 60.0, *SD* = 13.5), *t*(74) = -3.86, *p* < 0.001, *d* = 0.886, *strong effect*, and in the game, the HTH (*M* = 73.7, *SD* = 9.1) scored higher than the LTH (*M* = 63.7, *SD* = 10.4), *t*(74) = -4.37, *p* < 0.001, *d* = 1.023, *strong effect*.

## Discussion

The purpose of this study was to expand the process of tactical assessment of soccer players through the development of a video-based test based on the core tactical principles of play. The TacticUP video test for soccer displayed adequate content, construct, and face validity and was also a reliable measure of perceptual-cognitive and decision-making skills in soccer. In regard to validation, we used an expert panel to ensure content validity of the TacticUP video test for soccer, following previous validation studies of video-based tests (Roca et al., 2011; Larkin et al., 2014). Resorting to the opinion and feedback of expert practitioners is an important step to ensure that the instrument measures what it was designed to measure and represents the context and content of the domain being measured (Sireci, 1998). Furthermore, following recommendations from Larkin et al. (2014), we resorted to experts from four different countries, who provided a cross-cultural perspective of the game and were less likely to display any biases regarding a single “point of view” about soccer tactics from a particular country or culture, which is a novelty provided by our study when compared to other video-based tests.

The construct validity has usually been verified according to the power of distinguishing players from different performance contexts or with distinct accumulated amounts of training (Mangas, 1999; García-López et al., 2013; Serra-Olivares and García-López, 2016). In this regard, previous studies comparing individuals with different levels of expertise reported differences in their perceptual-cognitive and decision-making skills in soccer (Mangas, 1999; Roca et al., 2012), which endorses the construct validity of these tests. In our study, we found that players were distinguished based on their expertise levels in most of the core tactical principles, as well as in the offensive and defensive phases, and in the game. An important find that must be highlighted was that the offensive, defensive, and the game indexes presented a strong effect, which means that the expertise level of the players highly influences these variables. All differences mentioned above indicated that players with higher accumulated hours of training had greater perceptual-cognitive and decision-making skills in soccer compared to those with less training hours, which provides evidence of the construct validity of the TacticUP video test for soccer.

However, for some tactical principles, no differences were found between expertise levels. Interestingly, four out of five of these principles that did not display differences are performed inside the center of play (actions near the ball), which are contents usually learned early (around the age of 10) on sports development compared to those performed outside the center of play (actions distant from the ball) (Teoldo et al., 2017). Therefore, players of different groups of expertise might have accumulated sufficient stimuli regarding the principles performed inside the center of play earlier in their sport development, which may be the cause for the absence of differences for these principles.

The results also showed that there were more differences for the defensive than for the offensive principles. These results are probably related to the earlier acquisition of offensive instead of defensive tactical knowledge (González-Víllora et al., 2010). Thus, players who accumulated more training hours are probably more likely to display greater differences in their defensive perceptual-cognitive and decision-making skills once it is fostered late in sports development.

Additionally, our findings provide empirical support to theories that propose that improvement of perceptual-cognitive skills and knowledge occurs according to specific and accumulated practice in training (deliberate practice) (Anderson, 1983; Ericsson et al., 1993; Williams, 2000; Starkes et al., 2004). Another advantage of our test is that it is able to identify groups with different expertise levels based on objective measures (accumulated training hours) instead of only subjective ones (e.g., coaches’ assessment of players’ abilities), as suggested by literature (Blomqvist et al., 2000; Ericsson et al., 2006). In future studies, it would also be interesting to use a combination of both measures (objective and subjective) as it was shown to be a more robust method to create distinct groups of expertise (Sieghartsleitner et al., 2019).

Another important process for developing a test is face validity, which is related to the individuals’ acceptability of the test in order to ensure that results are an outcome of their engagement and effort with the task (Anastasi, 1988). However, other similar tests did not show values of this criterion of validation (Mangas, 1999; Elferink-Gemser et al., 2004; Roca et al., 2012; Serra-Olivares and García-López, 2016). In this study, face validity was measured based on the acceptance of the test by the participants, as suggested by Anastasi (1988). A relevant acceptance was reported, as 94% of participants enjoyed taking the test (Table 1). Moreover, players also showed a relevant inclination to retake the test (83.9%) in the future, which is a very important property of the test, since it allows coaches and researchers to assess players’ decision-making and perceptual-cognitive skills acquisition throughout the sport development process.

Reliability assessment is also a key point in the development of an instrument in order to inform whether a change observed in participants’ performance is a result of a training program/intervention or of an unreliable test. In our study, we showed appropriate values of reliability based on Landis and Koch (1977) references and compared them to those from previous validations of video-based instruments (Larkin et al., 2014). Therefore, the TacticUP video test for soccer may represent an important application in terms of reliably to track the development of perceptual-cognitive and decision-making skills in soccer, in training programs or school contexts. It can also be used to measure or compare the effectiveness of intervention programs that seek to develop individuals’ decision-making and perceptual-cognitive skills.

We would like to acknowledge that the TacticUP video test for soccer used the same theoretical background of the FUT-SAT (Teoldo et al., 2011b), which scientific knowledge has been produced since 2009. Among the studies carried out with FUT-SAT, there are empirical data that support the important role and relationship of tactical skills with: (i) sport development according to age (Teoldo et al., 2010; Américo et al., 2016; Borges P. H. R. et al., 2017); (ii) relative age effects, which are (dis)advantages between players born in different quartiles within a sport season (Teoldo et al., 2010b; Machado and Teoldo, 2016); (iii) players’ positional role (Machado et al., 2016; Rechenchosky et al., 2017a); (iv) training intervention programs (Souza et al., 2014; Aquino et al., 2015); (v) affective decision-making (Gonzaga et al., 2014; Andrade et al., 2016); (vi) training constraints (e.g., numerical superiority) (Bredt et al., 2016; Padilha et al., 2017); (vii) motor and technical skills (Praça et al., 2015; Aquino et al., 2016); (viii) peripheral perception (Gonçalves et al., 2017); (ix) maturation (Borges P. et al., 2017; Gonçalves et al., 2017); and (x) mental fatigue (Kunrath et al., 2018). Therefore, both TacticUP video test and FUT-SAT measure complementary constructs of tactical concept (i.e., perceptual-cognitive skills and perceptual-motor skills) based on the same theoretical and pedagogical foundations (i.e., the core tactical principles). Therefore, the use of such complementary instruments allows the design of more theoretically driven studies. Hence, these instruments should be used together in the future in order to test this assumption based on empirical data and measure the relationship between both types of skills in different phases of players’ sports development.

We overcame limitations from previous video-based tests by introducing: (i) the assessment regarding situations in which the observed player is not in possession of the ball in both offensive and defensive phases and (ii) the core tactical principles in its conceptual design, which provides transferability to the training process based on previous research (González-Víllora et al., 2015). This represents a step forward in the pursuit of correspondence between training and tactical assessment (Gréhaigne and Godbout, 1998). Furthermore, our test enables tactical assessment on a regular basis, which is key to qualify the process of talent identification and development over the years of sport development of a player (Sarmento et al., 2018).

Moreover, a characteristic of the test is the distinction between scores obtained in different core tactical principles assessed, which has more applicability and transference to training, in contrast to general assessments of offensive and defensive phases (González-Víllora et al., 2015). The results, based on the scores for each tactical principle, enable researchers and coaches to gather more specific information about players’ perceptual-cognitive and decision-making skills in distinct situations of management of playing space. This organization of information shows a more detailed picture of players’ strengths and weaknesses, allowing for more specific and individualized training interventions based on these results (Teoldo et al., 2011a).

The procedure used to assess the players in this test is based on their response selection, which enables the measurement of their decision-making skills based on the core tactical principles (Macmahon and Mcpherson, 2009; Teoldo et al., 2017). Another possibility afforded by the test is its joint use with verbal reports, which enables players to verbalize their thoughts (e.g., “why” an individual chose a given answer) in order to understand in detail the cognitive processes involved in tactical knowledge acquisition and development (Ericsson, 2006; Macmahon and Mcpherson, 2009). It can also be used with tools that objectively measure players’ eye movement, which may provide information on how individuals read the game and perceive the environment. It could provide data on what are the most important sources of information (e.g., player in possession of the ball, teammates, opponents) that enable players to choose the most appropriate responses (Williams et al., 2017).

Furthermore, the age of the individuals must be taken into account in order to use assessment tools in line with the stages of sports development (González-Víllora et al., 2015). We advise to start the use of the TacticUP video test for soccer with groups around the age of 11 years since the teaching of the core tactical principles ought to start around this age – when players are undergoing the final stage of their cognitive maturation and are able to use abstract thought to operationalize this category of tactical principles (Piaget, 1964; Teoldo et al., 2017). In addition, it would be interesting to investigate what type of soccer activities (e.g., deliberate practice, deliberate play, or competition) favors the development of decision-making skills and tactical knowledge in different age groups in order to seek empirical data to support the construction of a longitudinal soccer syllabus.

It is also important to acknowledge that players with higher levels of expertise in sports have a superior game reading skill and respond quickly to game demands (Mann et al., 2007; Hartigh et al., 2017). A meta-analysis on perceptual-cognitive expertise in sports carried out by Mann et al. (2007) showed that players with a superior level of expertise usually respond 35% quicker than their less skilled counterparts. Therefore, we suggest that the assessment of response time should be incorporated into this type of assessment used in our study as it provides information about the time required for a player to read the game in different situations, and it is related to higher levels of expertise in sport.

A limitation of this study regards the fact that the TacticUP video test for soccer was not taken by inexperienced soccer players, as was the case in previous validation studies (García-López et al., 2013; Bennett et al., 2019). Additionally, the sample of this study was comprised of only players who participated in competitions at the national level for their age group, which makes it not possible to make comparisons among different competitive levels. The use of inexperienced players could have enabled comparisons between groups (experienced vs. inexperienced players) with greater contrast. Furthermore, the inclusion of players from different competitive levels could be a more objective method to differentiate players regarding their expertise level (García-López et al., 2013; Bennett et al., 2019). Another possibility is to resort to information related to the quality of the training activities in which players were engaged (based on the microstructure of the training regime) rather than on the amount of training alone, so as to enable the categorization of expertise levels. Hence, we recommend that future studies address these comparisons between experienced players and groups with no previous experience in soccer and players who participate in different competitive levels (e.g., regional, national, and international competitions).

## Conclusion

We conclude that TacticUP video test for soccer followed the main steps for validation suggested by literature and presented adequate results for content, construct, and face validity, in addition to being a reliable measure. It implies that the instrument enables objective measurement of players’ strengths and weaknesses in the perceptual-cognitive and decision-making skills according to the core tactical principles of play. Moreover, the aim of this study was achieved, and we expanded the process of tactical assessment with an instrument that complements another available instrument (FUT-SAT). This type of approach can potentially improve future practical applications by soccer practitioners as well as the quality of research, as it aligned theoretical assumptions and enabled the link between different instruments, which allows for a more in-depth analysis of decision-making skills, perceptual-cognitive skills, and perceptual-motor skills.

### Practical Applications

1. This instrument can be used to measure the effectiveness of intervention and training programs on the development of perceptual-cognitive and decision-making skills of soccer players.
2. The test enables the measurement of the player’s perceptual-cognitive and decision-making skills based on the core tactical principles.
3. Development of soccer programs can benefit from this instrument as it allows reliable monitoring of players’ perceptual-cognitive and decision-making skills development.
4. The test can enable the creation of players’ tactical profiles based on their perceptual-cognitive and decision-making skills considering their strengths and weaknesses regarding the different core tactical principles.

## Data Availability Statement

The datasets generated for this study are available on request to the corresponding author.

## Ethics Statement

The studies involving human participants were reviewed and approved by the Ethical Commitee from the Universidade Federal de Viçosa. Written informed consent to participate in this study was provided by the participants’ legal guardian/next of kin.

## Author Contributions

GM contributed to the drafting and writing the manuscript. IC contributed to the developing the main concept of this research and drafting and writing the manuscript. Both authors contributed to the article and approved the submitted version.

## Conflict of Interest

The authors declare that the research was conducted in the absence of any commercial or financial relationships that could be construed as a potential conflict of interest. The handling editor is currently co-organizing a Research Topic with one of the authors IT, and confirms the absence of any other collaboration.
